# A Review of the Genomic Analysis of Children Presenting with Developmental Delay/Intellectual Disability and Associated Dysmorphic Features

**DOI:** 10.7759/cureus.3873

**Published:** 2019-01-12

**Authors:** Ramiah R Vickers, Jane S Gibson

**Affiliations:** 1 Genetics, University of Central Florida College of Medicine, Orlando, USA; 2 Pathology, University of Central Florida College of Medicine, Orlando, USA

**Keywords:** developmental delay, chromosomal microarray, genomics, uncertain significance, whole exome sequencing, intellectual disability, variants, next generation sequencing

## Abstract

This review describes the clinical criteria of developmental delay (DD)/intellectual disability (ID) as well as the various techniques that are currently implemented to diagnose neurodevelopmental disorders that typically present with associated dysmorphic features such as Angelman syndrome, Prader-Willi syndrome, and DiGeorge syndrome. These analyses include various forms of chromosomal microarray (CMA), which have proven to be superior to previously implemented techniques such as G-banded karyotyping and fluorescent in situ hybridization (FISH) analysis, as well as whole exome sequencing (WES), which is implemented as a secondary examination when CMA analysis is unrevealing. The clinical significance of identified variants and how it relates to facilitating the management of specific genetic disorders such as the above mentioned is also discussed. In addition, the importance of genomic databases and bioinformatics technologies as they relate to variant classification is also considered. Essentially, the discovery of pathogenic variants allows for enhanced management of a patient’s clinical phenotype, whereas the identification of variants of uncertain significance (VUS) has proven to have an increase in the number of associated conflicts as they typically generate more ambiguity in regard to the clinical manifestations present within the child. As a result, additional procedures need to be implemented to mitigate the issues that surround their identification. The concluding remarks are in regard to both the ethical and legal considerations of genetic testing as they relate to informed consent, testing of minors, how to handle secondary findings, as well as the anticipated future direction of genomic analysis.

## Introduction and background

Substantial delays in two or more aspects related to developmental progress can be utilized to describe developmental delay (DD). These aspects include gross and/or fine motor relating to movements of large and small muscles, respectively, cognitive function, language/speech, social, and lastly, activities of daily living that comprise actions related to personal hygiene/grooming, and dressing oneself. The presence of DD becomes evident when a child has failed to successfully exhibit achievement of developmental milestones associated with a specific age group. Thus, the term expresses that the child has potentially considerable deficits related to functional and adaptation abilities, which is ultimately suggestive of intellectual incapacity and disability later in life [1-2]. In this regard, the phrase DD is reserved for children younger than five years of age, whereas intellectual disability (ID) is practical for individuals older than seven years of age when IQ testing is more adequate and dependable [1,3-4]. In the United States, ID, which is utilized to substitute the term "mental retardation", is diagnosed by two systems: the American Association on Intellectual and Developmental Disabilities (AAIDD) and the fifth edition of the Diagnostic and Statistical Manual of Mental Disorder (DSM-5). The DSM-5 denotes that intellectual disabilities are neurodevelopmental disorders that commence in early childhood. These disorders are characterized by intellectual difficulties as well as struggles related to conceptual, practical and social aspects of living. Specifically, these individuals will have deficits in functioning such as perceptual and quantitative reasoning, verbal comprehension, abstract thought, comprehending instructions and rules, memory, problem-solving, and learning from experience [2]. Ultimately, previous investigations have established that DD/ID occurs in approximately 3% of the population and can be deconstructed into four classifications: mild, moderate, severe, and profound.

Mild ID accounts for approximately 85% of all cases. These individuals have an IQ between 50 and 70 and exhibit delays in regard to tasks of everyday living, conceptual development as well as proficiency in social settings. It has been reported that the mean IQ is approximately 100 and thus, persons who present with DD/ID are two standard deviations below the average, as they present with IQs below 70 [2,5]. Individuals with mild ID possess the ability to learn and retain knowledge related to the execution of duties of everyday life. This permits them to function with minimal assistance that is typically essential in regard to transitions and episodes of hesitation [2]. On the other hand, moderate ID accounts for approximately 10% of the population of people with ID. These individuals have an IQ range of 30-49 and have the capacity to learn rudimentary skills with regard to personal health and safety [2,5]. Therefore, individuals require limited assistance in their daily lives and can live independently in an environment such as a group facility in which they are able to receive moderate aid. Furthermore, the presence of mild or moderate ID will usually be exposed during the individual’s early educational years, as this signifies when their struggles in regard to academic learning become evident [2]. The sooner the medical intervention, the more probable the possibility to enhance the child’s proficiency in situations that necessitate adaptation. Recent analyses have also observed that individuals presenting with either mild or moderate ID are less likely to possess associated medical conditions in contrast to individuals with severe to profound ID [2,5].

Severe ID was determined to affect approximately 3.5% of all patients presenting with intellectual disabilities, and they generally exhibit significant delays related to developmental progression. Their IQ range is within 20-35 and they possess the ability to learn very simple routines which include a contribution to activities related to basic self-care customs. Moreover, these individuals generally have the capacity to comprehend speech; however, they have restricted communication skills [2]. In this regard, they require assistance for everyday activities, aid in social settings as well as supervision for their own safety. Finally, profound ID encompasses approximately 1.5% of total cases. They present with an IQ <20 and often have associated congenital conditions [2,5]. In this regard, they typically exhibit an extremely limited proficiency in regard to oral communication as well as various physical limitations. These individuals cannot live alone, thereby requiring around-the-clock assistance for daily activities that include careful supervision for their own safety. Ultimately, both severe and profound ID can be recognized within the first two years of life and frequently have accompanying medical conditions [2]. With respect to the population of persons presenting with ID, 30% to 40% of cases are correlated with a genetic origin, typically being chromosomal abnormalities [5]. These genetic sources for DD/ID can be either non-syndromic or syndromic, indicating that there are additional neurological conditions or associated dysmorphic features.

Dysmorphology

Essentially, dysmorphology is defined as the analysis of human malformations. Dysmorphic features are those that are observed in less than 5% of the population and they develop via one of the three mechanisms: malformations, deformations, and disruptions. A malformation is described as a structural defect that arises from an irregular developmental process, whereas a deformation is an atypical structure that develops as a consequence of non-disruptive mechanical influences to a formerly ordinary feature [1]. Lastly, a disruption results subsequent to the damage of a previously standard feature. Self-correction is typically never observed in circumstances of malformations and disruptions, not even in situations that were facilitated via surgical intervention. By contrast, the bulk of deformations possesses the potential to be amended with non-surgical methods of intervention [1]. 

Dysmorphic features can be further deconstructed into major and minor anomalies. Major anomalies are those that drastically alter the physical appearance and function of the patient and thus are evident upon birth. This division of anomalies usually includes orofacial clefts such as cleft lip/cleft palate (split in lip and roof of mouth, respectively), neural tube defects such as anencephaly (absence of cerebral hemispheres of the brain) and spina bifida (abnormality of the spinal cord) as well as limb deficiencies [6]. An additional major anomaly that is observed in clinical practice is omphalocele, which occurs as a consequence of an abdominal wall defect. It is characterized by the infant’s intestine or additional abdominal organs protruding from the umbilicus, usually enclosed merely by a delicate sheet of tissue. On the contrary, minor anomalies are of no significance to the functional or physical aspects of the affected individuals. Examples include hypopigmented patches, central incisor, single maxillary tooth, neck webbing, and wide-spaced eyes. Reports have documented that approximately 70% of minor anomalies exist within the head/face and hands [1]. Essentially, children discovered to possess dysmorphic features will often possess a distinctive arrangement of minor anomalies that generate a phenotype that deviates from what is considered to be standard. The presence or absence of specific clinical features including microcephaly, dysmorphic features, congenital anomalies, and seizures can facilitate decision making with regard to the appropriateness of genetic testing [4,6]. Ultimately, the assessment for dysmorphic features should occur in either one of two circumstances: when there is the presence of an obvious minor anomaly and/or when there is the presence of DD/ID [1]. This evaluation should include the descriptions of craniofacial features such as the eyes (shape, distance, etc.), mouth (cleft lip/palate), jaw (size and appearance), neck (webbing), trunk area (scoliosis), genitalia (structure), and the extremities (range of motion and number of digits), as it is recommended that any child presenting with DD/ID undergoes assessment focused on the discovery of traits that exhibit significant anomalous structural development. If laboratory results are ambiguous or there is the presence of a major anomaly, the current recommendation of the American College of Medical Genetics (ACMG) to the clinician is to refer the patient to a clinical geneticist for diagnostic examination [1].

Generally, there are three key motives to consider genomic examination: developmental disorder, growth disorder, and congenital anomalies, which are structural and/or functional alterations that occur during intrauterine life. With this in mind, the intent of genetic testing is to hopefully provide the patient and their families with either a clinical or an etiologic diagnosis. Specifically, a clinical diagnosis can be converted into beneficial medical knowledge in regard to prognosis, recurrence risk, and forms of existing therapy for the specific condition [1,7-9]. An etiologic diagnosis signifies that there is adequate literature evidence to make a causal correlation with the patient’s phenotype and a particular disorder of interest [1]. Essentially, the overall examination of the patient should consider the child’s medical history as well as the family’s history, including both neurological and physical examinations, as well as behavioral analysis to identify any apparent characteristics that may indicate a particular syndrome or disorder. The primary inspiration for genomic evaluation in children presenting with DD/ID is to diminish ambiguity, due to the reality that ID is often sporadic and thus lacks evident environmental or familial influences [10]. Previous studies have established that structural chromosomal alterations have the ability to trigger an assortment of clinical manifestations including DD/ID, dysmorphic features and congenital anomalies [1]. Ultimately, in 2010, the ACMG recommended that chromosomal microarray (CMA) be implemented as the first tier of genomic analysis for patients presenting with DD/ID [11].

## Review

Chromosomal microarray (CMA)

CMA refers to a type of microchip-based technique that permits the automated and simultaneous analysis of numerous sections of DNA. It relies on both molecular and cytogenic knowledge to assess thousands of loci throughout the human genome for chromosomal abnormalities such as microdeletions and microduplications [1,11]. In general, CMA is utilized to identify genomic abnormalities associated with numerous developmental disabilities such as DD/ID as well as associated congenital malformations and cognitive and behavioral abnormalities [11]. It has been previously reported that CMA analysis has a diagnostic yield of approximately 20% to 25% for which it is especially beneficial for detection of factors that are possibly indicative of chromosomal imbalances such as variations in DNA copy number. However, it will not detect balanced copy number changes such as inversions/insertions and reciprocal translocations, as these rearrangements seldom (< 1% of cases) disrupt a gene leading to the development of a clinical phenotype [1,3,5]. These modifications in DNA copy number are known as copy number variants (CNVs). Furthermore, computer analysis is also utilized to compare the patient’s genetic material to that of a reference sample which is utilized as a "standard" control. Dissimilarities between a control sample and a patient sample are referred to as "variants". It has been previously reported that the frequency of disease-causing CNVs is highest in children with moderate to severe ID that is also associated with dysmorphic features [5]. In relation, impairment and accompanying characteristics that are highly severe will result in an augmented probability in regard to the identification of a causative CNV, thereby permitting a higher diagnostic yield in comparison to patients with mild ID [5-6,11]. Although the majority of identified CNVs develop in a sporadic fashion, others have the potential to be inherited from a parent resulting in a recurrence risk as high as 50%. These intermittent modifications are referred to as *de novo* (new) mutations or variants. It has recently been established that more variants have been reported in persons presenting with DD/ID than in healthy controls [10]. In addition, these variants have been repetitively discovered in many neurodevelopmental disorders subsequent to CMA analysis.

Essentially, there are currently two forms of CMA that are implemented in the genomic analysis; these being, array comparative genomic hybridization (aCGH) and single nucleotide polymorphism (SNP) array, both of which have been confirmed to be valuable for the detection of CNVs such as microduplications and microdeletions. aCGH has been previously established to permit an etiologic diagnosis in 15% to 20% of cases with patients presenting with an unknown cause of DD/ID with or without associated dysmorphic features [5,12]. This is a reputable comparison to the mere 3% to 5% that would have been detectable by other means [12]. For this particular technique, "test" and control DNA samples are fluorescently labeled and subsequently united and applied to a microarray, which is essentially a network of DNA segments of identified sequence that is utilized to analyze and pinpoint target DNA fragments. The samples are single-stranded and will, therefore, attempt to hybridize to the single-stranded probes of the array. Subsequently, electronic imaging is utilized to analyze hybridized segments with regard to the quantification of the intensity of their relative fluorescence. This resulting fluorescent intensity is then linearly plotted for each chromosome and the ensuing proportions are then computed to yield data on the quantity of copy number changes in the "test" genome in comparison to the "reference" genome, thus permitting the identification of CNVs [12]. More specifically, in areas where there is equal hybridization of the test and control DNA, there will be an overlap of the fluorescent dyes. In areas where there is a deletion within the patient DNA, only the dye color of the control DNA will be visible as it does not have to compete to hybridize to the microarray. Finally, if there is duplication within the patient DNA, only the fluorescent dye of the patient DNA will be visible as it is able to out-compete the control DNA sample due to its overrepresentation.

On the other hand, SNP array can be implemented to array thousands of oligonucleotides for the exposure of all probable SNPs in the target DNA [13]. SNPs are basically a deviation of a single nucleotide and have been established to represent the most frequent type of variation within the human genome. The purpose of a microarray-based SNP analysis is to identify slight variations between whole genomes. Each individual has an abundant quantity of SNPs that have the potential to cause adverse effects if it falls within the coding sequence of a crucial gene [13]. These effects would arise if the SNP resulted in a revision of the amino acid sequence in such a way that would consequently impact the protein generated, thereby leading to the potential to cause an alteration at the phenotypic level. In this regard, this technique utilizes oligonucleotides that are arrayed to probes. A mixture of "test" and reference DNA samples are fluorescently labeled and will serve as the targets of interest. A piece of technology known as the "Gene Chip" possess allele-specific probes for approximately 1,500 SNPs and therefore each allele on the array correlates to a specific genetic locus [13]. Thus, upon hybridization and determination of fluorescent intensity, variations between the two genomes can be identified in a similar fashion to what was previously described utilizing aCGH.

In comparison to aCGH, microarray-based SNP analysis has the potential to detect instances of uniparental disomy (UPD), which is described as the phenomenon in which a child inherits two copies of either a maternal or paternal chromosome, instead of one from each parent. It also demonstrates augmented sensitivity to chromosomal mosaicism (as low as 5%) compared to aCGH [6,14]. Mosaicism can be defined as a condition in which cells within the same individual, that developed from a single fertilized egg, have a varying genetic makeup [6,11,15]. This type of genetic variation typically has numerous challenges related to its detection and therefore, consequently goes undiagnosed in the majority of cases. Ultimately, due to its enhanced sensitivity for detection of single base-pair variations within the genome, microarray-based SNP analysis has the capacity to identify previously undetectable cases of mosaicism. It has become progressively apparent that children with DD/ID and associated dysmorphic features present with fundamental genomic abnormalities that can be identified by either aCGH or SNP array [5-6,11,14]. Thus, this knowledge serves as the first evidence that CMA analysis should be implemented prior to conventional techniques such as G-banded karyotyping and fluorescent in situ hybridization (FISH).

G-banded karyotyping

A karyotype is described as a highly accurate, visual representation of the quantity and structure of chromosomes present within a cell. For this method, metaphase chromosomes are treated with trypsin that permits their partial digestion. Subsequently, the chromosomes are stained with Giemsa and regions of varying transcriptional activity will establish a distinctive sequence of bands. More specifically, chromosomal regions known as heterochromatin are gene poor (AT-rich) and will absorb the dye more readily and thus will produce dark bands. This is a direct result of these particular regions being less transcriptionally active and thus more condensed. Therefore, trypsin cannot digest these regions readily, and so they will take up more of the dye and be observed as darker bands. By contrast, areas known as euchromatin are gene-rich (GC-rich) and will be more transcriptionally active. Being in a less condensed conformation, these regions are more readily digested by trypsin, thereby producing lighter bands as a result of less dye absorption. Ultimately, as a result of varying gene transcription, each chromosome has its own distinct banding pattern [5-6]. Thus, karyotyping is a highly accurate technique for the identification of structural chromosomal abnormalities.

In this regard, karyotype analysis was previously the first approach to genomic analysis. Over the years, CMA has replaced G-banded karyotyping as the first-tier test in the genomic evaluation of children presenting with DD/ID and additional behavioral disabilities [1,11]. As the first evidence of why this transition occurred, CMA permits detection of CNVs at a substantially higher resolution in comparison to G-banded karyotyping. CMA offers a minimum resolution of approximately 100 kb compared to the 3-5 Mb required for karyotyping [14]. This ultimately demonstrates a 30 to 50-fold increase in the resolution [6]. Additionally, CMA has proven to be more sensitive than karyotyping which has a diagnostic yield of approximately 3%. Thus, CMA techniques are significantly more effective than karyotype analysis in detecting genomic abnormalities that result in a disease state. Finally, CMA also exhibits the capacity to detect the vast majority of the chromosomal abnormalities identified via a standard karyotype. Therefore, it is advised that G-banded karyotyping be reserved for obvious chromosomal abnormalities such as Down syndrome/trisomy 21 [14].

Fluorescent in situ hybridization (FISH)

Likewise, FISH analysis has also existed as a top-tier test for the identification of chromosomal abnormalities. This technique utilizes sequence complementarity to allow the adherence of fluorescently labeled probes to specific chromosomal regions. These probes are prepared as short, single-stranded DNA fragments that are complementary to the gene of interest. Therefore, when the probe attaches to its partner sequence, fluorescence microscopy can be utilized to record its position within the genome. In this regard, FISH permits the identification of present and/or absent DNA sequences within a chromosome. Recent reports have established that when utilized for children with neurodevelopmental disabilities, telomere FISH analysis has generated a significantly higher diagnostic yield in comparison to conventional FISH analysis [4,11,14]. Nonetheless, all FISH analyses are restricted by their “resolution, color perception of the human eye as well as by the logistics of the probe design” [16]. Specifically, the implementation of FISH is limited to cases in which the patient’s phenotype is indicative of a definite disorder such that the precise probe can be utilized. This is a result of probes adhering to location-specific regions within the genome for which they have a complementary sequence. Therefore, the medical professional must have a clue as to what condition the patient may have so that the proper probes may be utilized. 

CMA tackles this matter by utilizing a computer-based approach to quantify the fluorescent signal in addition to hybridizing the free DNA from the patient to individual probes rather than the contrary [1]. Therefore, the genetic sequence is not required to be known with CMA analysis as is the case when utilizing FISH. Metaphase chromosomes for FISH analysis are sufficient for the detection of deletions; however, this method fails to detect duplications. This occurs because two duplicates at adjacent locations result in overlapping fluorescent signals which go undetected [11]. Ultimately, CMA is less expensive and has higher diagnostic yields than both G-banded karyotyping and FISH analysis. CMA also detects the subtle abnormalities that may be overlooked by standard chromosomal analysis. Therefore, it has proven to be superior for the identification of syndromes associated with DD/ID such as Angelman syndrome, Prader-Willi syndrome, and velocardiofacial/DiGeorge syndrome (VCFS/DGS).

Specific genetic conditions

Angelman syndrome is characterized by DD/ID, severe speech impairment, frequent laughter and excitability, fascination with water, hand-flapping, seizures, and difficulties with feeding. It is also associated with dysmorphic features such as hypopigmentation in the skin and eyes, scoliosis, and strabismus (cross eyes). Angelman syndrome has been determined to affect approximately 1:12,000 to 1:20,000 births, with 70% of cases arising from a maternal deletion on chromosome 15q11-q13 [1,17]. The gene of significance for this condition is UBE3A, which encodes for ubiquitin-protein ligase E3A that has a critical responsibility in the function and development of the nervous system [17]. This protein has been discovered to tag other proteins with ubiquitin which allows them to be recognized and obliterated by the proteasome. This process, therefore, eliminates damaged and/or unnecessary proteins, resulting in the preservation of normal cell function by regulating the equilibrium of protein synthesis and degradation at the synapses between nerve cells, where cell-to-cell communication transpires [17]. Thus, it is deemed that the absence of UBE3A results in the characteristic features of Angelman syndrome. Furthermore, approximately 10% of cases can be attributed to UPD where the patient inherits two copies of their paternal chromosome 15 and no copy from the mother. This is problematic because the UBE3A gene on chromosome 15 undergoes a phenomenon known as genomic imprinting, which results in parent-specific activation of certain genes [17]. This describes the process in which maternal and paternal chromosomes are marked with what is known as "imprints". These imprints are essentially methyl groups that serve as epigenetic markers and do not alter the specific genetic sequence. Generally, the paternal copy will be methylated, while the maternal copy remains un-methylated [17]. This is significant because neurons exclusively express the maternal allele, while the paternal allele is in a dormant state [17]. Therefore, if a patient inherits two copies of the paternal allele, as in cases of UPD, they will not possess an active form of UBE3A within the brain and thus will present with Angelman syndrome [17]. Likewise, Angelman syndrome may also develop from imprinting defects in which the maternal copy possesses a paternal imprint and is thus not activated within the neuronal cells. 

Prader-Willi syndrome is characterized by DD/ID, obesity/rapid weight gain, hyperphagia, infertility, feeding problems, and temper tantrums. Additionally, it is associated with dysmorphic features such as hypopigmentation, small hands, and feet, as well as strabismus and it was determined to affect approximately 1:10,000 to 1:30,000 births [1]. In 70% of cases, Prader-Willi syndrome arises from a paternal deletion on chromosome 15q11-q13, thus making it contiguous with Angelman syndrome which arises from the same deletion but on the maternal chromosome. Essentially, one of the genes within the deleted region is UBE3A-ATS which encodes for the antisense DNA strand of the protein UBE3A. During the process of genomic imprinting, it is believed that this gene is exclusively expressed on the paternal chromosome 15 in neurons [17-18]. Thus, it is hypothesized that it directly mediates the suppression of paternal UBE3A within the brain [18]. Deletion of this gene on the paternal chromosome is believed to be the causative effect of the resulting Prader-Willi syndrome. Additionally, Prader-Willi syndrome can arise from UPD in which the patient inherits two copies of their maternal chromosome 15 which accounts for approximately 10% of cases. 

VCFS/DGS is characterized by approximately 180 physical and behavioral clinical features including DD/ID, hearing loss, immune deficiency, psychiatric illness, congenital heart anomalies (70% of cases) such as Tetralogy of Fallot (15% of cases), and cleft palate [1,19]. Specifically, Tetralogy of Fallot is characterized by skewed development of the AP septum that results in inadequate blood circulation to the lungs, whereas cleft palate refers to a congenital split on the roof of the mouth. 75% of patients also present with a hypernasal speech that can be correlated to an anatomic dysfunction of the soft palate that results in an abnormal resonance within the voice [19]. The associated immune deficiency usually leads to recurrent respiratory infections in early childhood [19]. Although most patients will not develop a psychiatric disorder, they are at a 25-fold risk increase for the development of attention deficit disorder, schizophrenia, and bipolar disorder compared to the general population [19]. VCFS/DGS typically presents with dysmorphic features such as cleft palate, ocular hypertelorism, vertebral anomalies, as well as strabismus and its prevalence is approximately 1:4,000 births [1]. Due to the complexity of this condition, no case has been reported, wherein the patient possessed even most of the associated clinical phenotypes. Generally, a 3-Mb deletion on chromosome 22q11.2 is identified in 93% of individuals with VCFS/DGS [19]. On the other hand, a 1.3-Mb deletion is reported in approximately 7% to 8% of individuals [1]. These deletions contain approximately 40 and 20 genes, respectively. Moreover, previous reports have established that 93% of total cases are *de novo* and can be passed on to future offspring in an autosomal dominant manner [1,19]. As a result of such sizeable deletions, these patients will present with ailments that affect multiple organs throughout the body. Therefore, these patients will typically have medical problems that persist in infancy and early childhood [1,19]. During their early educational years, difficulties relating to cognitive and behavioral learning will become evident. Psychosis is generally subtle within these patients, but when reported, it is documented to commence in late adolescence, continuing into the patient’s adult years. Ultimately, CMA analysis has proven to be the superior technique for detecting the previously mentioned syndromes along with countless others. Occasionally CMA analysis can be normal although the patient presents with syndromic characteristics. As an illustration, for the previously mentioned conditions, approximately 10% to 15% are of an unidentified origin. Therefore, in these circumstances, the ensuing step in the diagnostic process would be to execute whole exome sequencing (WES).

Whole exome sequencing (WES)

WES can be utilized to identify a causative genomic variant in an individual presenting with DD/ID with associated dysmorphic features for whom conventional testing such as CMA was unrevealing. The process refers to the sequencing of the protein-coding regions of the human genome, defined as the exome. WES is a type of next-generation sequencing (NGS) technology that sequences millions of small fragments of DNA in parallel [20]. During WES, each of the bases is sequenced multiple times as a way to guarantee the delivery of highly accurate data. Subsequently, bioinformatics analyses are utilized to unite the resulting fragmented segments by pinpointing the individual reads to the human reference genome [20]. Thus, WES provides extensive and high-resolution detection of genetic variants as well as augmented sensitivity that allows for the identification of genomic variations that are generally more challenging to detect, such as mosaicism [20-21]. As a result, WES analysis can facilitate determining the causative effects correlated to a particular condition, thereby inspiring novel therapeutic strategies to facilitate with the management of the patient’s symptoms. WES has proven to be a superior technique when compared to conventional sequencing methods such as Sanger sequencing.

Sanger sequencing functions by annealing a primer to the template strand of DNA, which is the strand that is to be sequenced. Deoxyribonucleotides (dNTPs) are the building blocks; however, the aspect that makes this method successful is the incorporation of dideoxriboynucleotides (ddNTPs), which are fluorescently labeled with a different dye color for each of the four bases to permit simple visualization. Specifically, ddNTPs lack the 3’OH group that is necessary for construction of the phosphodiester bond that unites adjacent nucleotides. Thus, DNA polymerase is incapable of continuing on with the incorporation of additional nucleotides, thereby resulting in a truncated DNA fragment upon addition of a ddNTP within the sequence. Subsequent to an extension, the products are subjected to separation via capillary electrophoresis, in which the sample is injected into a glass capillary packed with a gel polymer. An electrical current is then applied to promote migration of negatively charged DNA fragments towards a positive electrode. The smaller fragments migrate quicker because they do not encounter as much resistance as the larger fragments, thereby allowing them to travel through the medium more effortlessly. As a DNA fragment passes through the base end of the capillary, a laser will serve to excite the fluorescent dye attached to the fragment. This excitement will result in the emission of light that will be detected by an associated sensor. Ultimately, the end result of this process will be a color-coded electropherogram that is representative of the sample of DNA fragments that has been separated by a base.

By contrast, NGS techniques have demonstrated their ability to capture a wider range of variation than previous sequencing techniques. They are characterized by their ability to analyze enormous quantities of DNA in parallel, thereby providing considerably more knowledge at competitive rates [22]. Sanger sequencing is limited by the fact that it relies on primers that must attach to specific target sequences on the DNA strand. Multiple reactions are also required to achieve the entire sequence of a gene of interest as a consequential result of the primers only being dependable for small fragments of the gene. Likewise, Sanger sequencing is restricted to the identification of substitutions as well as small deletions and insertions [20]. In reality, most conventional sequencing technologies will miss the identification of larger duplications and deletions, which is one of their chief shortcomings. This especially occurs when, for instance, a deleted segment includes the expected binding site of the primer. Ultimately, all of the previously mentioned facts provide sufficient evidence for implementing NGS technologies such as WES over conventional sequencing techniques such as Sanger sequencing.

WES has proven to be quite successful for the detection of variants, especially in cases that previous CMA analysis did not provide a beneficial diagnosis. WES requires less input DNA and therefore has the capacity to be more efficient than microarray-based methods [23]. However, some studies have observed that CMA techniques have superior SNP detection in comparison to WES, which is why it is implemented as a secondary testing method [23]. For the process of WES, DNA samples are fragmented prior to selective hybridization of oligonucleotide probes to the target regions of the exome. Magnetic beads are then utilized to adhere to the probes, thereby permitting the consequent elimination of the non-protein coding regions which are not the focus of the analysis. Subsequently, PCR is utilized to amplify the resulting sample prior to the sequencing process which is ultimately followed by bioinformatics analysis to assist with variant detection. Identified variants are classified into one of five categories: pathogenic, likely pathogenic, uncertain, likely benign, or benign [24]. Additionally, variants of uncertain significance (VUS) can be further deconstructed into three categories: favor pathogenic, uncertain/ambiguous, and favor benign [9]. Following genomic testing, the clinical significance of the discovered variants must be determined. This essentially denotes determining how the identified variant correlates to a particular disease or associated phenotype. The clinical significance of identified genomic variants can be established by answering three crucial questions: “1) Does the variant modify the function of the affected gene, 2) Can the resulting functional modification result in disease or a specific phenotype?, and finally, 3) Is the associated disease or phenotype pertinent to a specific clinical disorder present in the affected individual?” [25]. Ultimately, population, functional, and statistical evidence, as well as inheritance patterns and implicated proteins, all need to be carefully considered with regard to facilitating the determination of whether or not an identified variant can be correlated to a specific clinical phenotype [9,21].

Classification of variants

CNVs specifically are classified as being either benign or likely benign because they are typically discovered within regions of the genome with very few coding genes and there is also insufficient literature evidence in support of potential pathogenicity [26]. Variants that are classified as either likely benign and likely pathogenic are classified as “likely” because there is a 90% or greater certainty that the variant is either benign or pathogenic [24]. Likewise, inheritance of a variant from an unaffected parent is indicative that the variant is either benign or likely benign and it is deemed to not be a causative effect of the patient’s condition. On the contrary, VUS can be discovered in a gene associated with a clinical condition in question; however, there is inadequate evidence that the variant is pathogenic [26-27]. Additionally, they can also be discovered in a gene of uncertain significance in a circumstance in which the nature of the variant is indicative that it may have an influence on the patient’s phenotype [27]. This particular situation is typically observed with *de novo* mutations. Variants are also categorized as having uncertain significance if there is a lack of evidence of pathogenicity in an acknowledged disease-causing gene that is not relevant to the patient’s phenotype [27]. It is valuable to acknowledge that depending on their precise location and orientation within the genome, CNVs possess the capacity to promote complex functional effects on the patient. Specifically, CNVs can potentially effect expression of a gene from a distance as well as effect adjacent genes by inserting into varying locations throughout the genome [26]. Ultimately, variants are classified as having uncertain significance when there is ambiguous or contradictory data associated with it; however, some evidence associated with the variant may lead toward an inclination of either pathogenicity or the variant being benign. It is to be expected that the more locations within the human genome that are analyzed, the more probable VUS are to be discovered.

In contrast to the above, variants that are classified as either pathogenic or likely pathogenic are not reported in healthy individuals. They generally are not inherited from a parent and therefore occur as *de novo* mutations. Most importantly, pathogenic variants are established to result in consequential adverse effects on either the function or expression of a critical gene that has been associated with a specific condition. As a result, patients identified as possessing identical pathogenic variants will present with similar clinical phenotypes, thereby permitting the establishment of sufficient evidence of pathogenicity within the related literature. A variant, specifically in regard to CNVs, is more probable to be classified as pathogenic if it is gene-rich, large in size, contains genes with associated clinical phenotypes, and if it coincides with a variant known to be correlated to a specific condition [6]. Additionally, if a variant is discovered to have a high frequency in the population, this can be utilized as evidence against it being pathogenic. On the contrary, a variant being absent or having a low frequency within the population cannot be utilized as evidence that the variant is pathogenic [9]. Ultimately, the determination of pathogenicity should encompass all evidence and occur independently of identifying the primary cause of disease within a patient such that an appropriate conclusion can be made [24].

Clinical management after the diagnosis

Once the clinical significance of a variant is established, the subsequent step would be to determine how to manage the knowledge obtained, as new diagnostic data will beneficially influence the management of patients with neurodevelopmental disabilities such as DD/ID. Novel information obtained from genetic testing which results in a clinical diagnosis will enable a clearly defined prognosis, more focused therapy programs, and clinical follow-up tailored to the patient’s needs [5,11]. This knowledge can also be utilized to provide knowledge with regard to recurrence risk for parents and families in addition to advising relevant care providers and school personnel such as nurses and classroom aids. Management strategies for neurodevelopmental disorders can be separated into three categories: 1) those that alleviate the primary cause of the ID, 2) treatment for associated comorbid mental and/or physical disorders for enhancement of the patient’s functioning abilities, and finally, 3) assistance focused on special education, rehabilitation as well as cognitive and behavioral interventions [2].

As an illustration, patients with confirmed Angelman syndrome will require regular developmental monitoring and early education planning. Due to their severe speech impairment, they should also undergo communication therapy focused on non-verbal cues including sign language and picture communication for younger children. It is recommended that patients also undergo behavioral therapy to facilitate with improving their short attention spans which will ultimately advance their developmental progress. Other aspects such as vision, feeding issues, and sleep disturbances should also be closely monitored. Likewise, any seizure activity is generally controlled with medication and reports of scoliosis should also be closely observed. Ultimately, these patients are expected to have a normal life expectancy, and as they mature, it is anticipated that they will become less excitable and their sleeping difficulties should improve [17]. However, their ID, speech impairment, and seizures will persist throughout their lives.

In relation, patients successfully diagnosed with Prader-Willi syndrome will need regular monitoring of their developmental progress, vision, sleeping patterns, and behavioral therapy to manage their temper. Due to associated hyperphagia, which is excessive over-eating and feeling of hunger, the patient’s food consumption should also be monitored closely as they have a propensity to become obese as a result. Therefore, it is recommended that the patient have a diet plan and exercise regularly to thwart the onset of obesity [17]. Feeding difficulties due to viscous saliva also necessitate regular observation as to discover and document any gastroesophageal problems that may arise. Additionally, studies have exhibited that administration of growth hormone has provided the benefit of reducing fat mass within these patients [1,17]. Prader-Willi syndrome also results in hypogonadism and infertility, and therefore these patients will not be capable of having biological children.

Patients with VCFS/DGS will require close surveillance of developmental progress in addition to surgical correction of congenital heart defects and facial anomalies. These individuals will also typically present with immune deficiency and should avoid live vaccinations. They also usually receive medication to facilitate with endocrine problems such as hypercalcemia [1,19]. Ultimately, the management for this particular syndrome is extremely complex and is thus tailored to the clinical phenotypes present within each patient.

Management of variants of uncertain significance

As just previously illustrated, when a patient is presented with a confirmed pathogenic variant, the diagnosis ultimately enables the implementation of a targeted treatment plan that is tailored to the specific condition. However, the management of identified VUS can be a bit strenuous. To begin, there are numerous questions that may arise; however, four main questions that clinicians need to address will be the focus here: “1) Should VUS be disclosed to patients? 2) How should the patient be counseled? 3) What follow-up studies should be implemented? and finally, 4) What should happen if and when the variant is re-classified”? [25]. Specifically, the ACMG recommends that a VUS should not contribute to facilitating a clinical determination [24]. Instead, advancements should continuously be made to reclassify the variant as either (likely) pathogenic or (likely) benign. Meanwhile, the patient should have routine monitoring of their clinical phenotypes. Some clinical geneticists believe that clinicians and other medical professionals without genetics expertise may fail to comprehend the importance of VUS, and therefore may also have inappropriate responses to their identification [25,27]. If this is accurate, and clinicians are indeed themselves misconstruing VUS, they may also be conveying that the variant is the causative agent in regard to the patient’s condition and this inappropriate response may lead to conflict later. VUS can be particularly difficult for patients and their families to process and can severely influence decision making. In this regard, a patient’s family should never in any circumstance harbor feelings of frustration, anxiety, or uncertainty which ultimately lead to misunderstanding of results. Thus, strategies to improve pre- and post-test counseling in regard to the identification of VUS need to be implemented. In this way, the patient’s family can receive maximum understanding and minimized adverse emotions [25]. Essentially, additional tactics need to be implemented to describe VUS in a way that both patients and clinicians without the expertise in medical genetics have enhanced comprehension to avoid potentially negative outcomes.

Conflicts have also been observed to develop specifically in regard to what should occur when a variant is reclassified. Currently, a suggestion is that it be the laboratory’s responsibility to distribute any updated reports to the clinician who is then responsible for updating the patient [25]. However, there are three core considerations that cause conflict in this regard: 1) How long was the time frame between the discovery of the variant and its subsequent reclassification? 2) Is the clinician still working with the patient? and finally, 3) Is pertinent contact information available for the patient so that they may receive any updated results? [25]. The nature of these questions is ultimately what creates problems surrounding the discovery of VUS. Typically, it will be some time before a variant is able to be reclassified. During this time, the patient, for various reasons, may no longer be under the direct supervision of the clinician who was present during the identification process. Furthermore, if the patient is under the care of a different clinician, it is crucial that they have relevant contact information on file with their previous clinician in the case that there is an update on the status of their VUS. Unfortunately, the reality is that many patients are unaware that their VUS had been reclassified [25]. This is most likely due to the usually significant periods of time between the time that their variant was identified and the time at which it was reclassified. Due to the previously addressed issues, the ACMG recommends that laboratories offer periodic inquiries to healthcare providers to provide updates on whether knowledge with regard to a previously classified variant, specifically those classified as having an uncertain significance, has changed [24]. Laboratories should also provide policies with regard to the reanalysis of data collected from testing and whether or not an additional cost will be associated [24]. Thus, the implementation and maintenance of genomic databases are crucial for this proposition to be effective.

Importance of databases

Essentially, databases contain a continuously growing number of recently discovered variants within the human genome. They serve the purpose of providing a central network for facilitating the interpretation of new variants discovered via diagnostic genomic testing [28]. Therefore, genomic databases are extremely beneficial for gathering and storing valuable data on previously discovered variants. There are several types of databases including population, disease-specific, and sequence databases. Population databases will be beneficial in the attainment of variant frequencies in large populations, whereas disease databases will primarily be composed of variants identified within a patient presenting with a specific disease as well as the assessment of the pathogenicity of the associated variant [24]. It is vital that these databases are constantly updated in order to facilitate the interpretation of identified variants and the possible reclassification of VUS. Examples of currently utilized databases include DECIPHER (Database of Chromosomal Imbalance and Phenotype in Humans using Ensembl Resources), DGV (Database of Genomic Variants), dbVar (Database of Genomic Structural Variation), the Matchmaker Exchange (MME), and ClinVar. 

DECIPHER is a disease-related database that permits public access to data in addition to password-protected logins for researchers and associated medical professionals to securely submit and manage patient information [14]. ClinVar is yet another public archive disease-associated database that comprises the interpretations of human genetic variants and their significance to an associated disease state [29]. DGV will provide relevant control data for assessments whose objective is to associate genomic variation with a specific phenotype [30]. dbVar also supports submission and organization of identified variants to facilitate researchers in the evaluation of genomic variations within the population [24,31]. Together, dbVar and DGV represent the most comprehensive archive of structural genomic variation in the entire world. These two databases also exchange knowledge regularly as a means to “maximize the representation of complex genetic disorders” [31]. In relation to exchanging data, the MME was launched to provide a network of linked databases containing knowledge in regard to rare genetic phenotypes through the implementation of a common application programming interface (API) [32]. As time passes, the clinical significance of VUS is often resolved as a direct result of gathering additional information and efficient utilization of genomic databases. With the assistance of these databases, the involved clinician and/or medical geneticist will eventually be able to make the determination of whether the VUS is pathogenic or benign. Most frequently, the VUS will be determined to be benign [25]. Ultimately, the true significance of genomic databases is to maintain updated information regarding classified variants, especially those of uncertain significance.

Bioinformatics 

A number of bioinformatics technologies may be implemented to facilitate the prediction of the potential impact of variants on the affected gene and associated protein. These technologies rely on alignments of computational derivation that suggest when a particular sequence is significant in regard to an affected gene and protein function [9]. Bioinformatics technologies are valuable for providing guidance; however, they cannot determine or rule out pathogenicity [9]. Essentially, these tools support five analytical steps: raw data quality assessment, pre-processing, alignment, post-processing, and variant analysis [33]. Raw data quality assessment will determine the need for any pre-processing steps. These pre-processing steps can include aspects such as the removal of redundant reads as well as contamination from primers. [33]. Following pre-processing, alignment serves to map "reads" to a reference genome, and it does this with high accuracy and efficiency. Specifically, the optimal alignment is established by utilizing an algorithm that is tolerant to regions where genomic variations may occur [33]. Due to the high data content produced by NGS technologies, these algorithms are required to align millions of reads at a sufficient speed. Subsequent to alignment, post-processing is implemented to minimize artifacts that have the potential to impact the quality of downstream variant calling [33]. This step can be deconstructed into indel realignment and base quality score recalibration (BQSR). Indel realignment will essentially improve the alignment quality of the target region. Likewise, during sequencing, a quality score is generated by the sequencer that is representative of the confidence of the data. These machine-generated scores have the tendency to be systematically biased and thus inaccurate [33]. Therefore, BQSR is implemented to enhance the accuracy of the confidence score prior to the final step. Finally, the last analytical step that is implemented is variant calling, in which the identified variants undergo a process of filtration and prioritization relative to a specific condition in question.

A major restriction of the currently implemented capture-based NGS techniques is that large duplications and deletions are often undetected due to NGS data being less accurate in regard to CNV identification [34]. Therefore, SeqCNV was developed as a more sensitive method to address this limitation. This tool can be utilized for the analysis of NGS data for the identification of pathogenic CNVs in human genetic disease. Essentially, this statistical method evaluates copy number ratio via a maximum penalized likelihood estimation (MPLE). Additionally, the identification of CNVs of varying lengths is permitted via a novel segmentation algorithm. Investigations of the SeqCNV technology have established that it provides numerous advantages when compared to traditional utilized methods such as paired-end mapping (PEM), which was restricted in regard to the read length, as well as the depth of coverage (DOC), which was associated with a high rate of false-positive results [34]. SeqCNV alleviates both of these issues with its ability to detect CNVs of varying sizes with a significantly lower rate of false positives. Despite this, it must be considered that the size of a CNV will affect the sensitivity of its identification. It is easier to identify larger CNVs as those of a smaller size can be difficult to distinguish from background [34]. A possible resolution would be to increase the depth of sequencing which would, in turn, enhance the overall efficiency of the SeqCNV technology [34]. Ultimately, NGS technologies generate numerous quantities of data that require specific technologies to facilitate data management, analysis, storage, and archiving [22]. Bioinformatics tools have proven to be extremely beneficial in efficiently managing the large quantities of data without error in addition to ensuring enhanced documentation and quality [22,33].

Ethical considerations of genomic testing 

There are numerous ethical considerations associated with genetic testing. One issue is that the testing may result in misinterpretation of results, thereby leading to feelings of guilt or blame, subsequently resulting in a potentially traumatic experience for the family. Therefore, appropriate pre-testing counseling is crucial to thwarting post-result conflicts [6,25]. Another dilemma relates to procedures that are necessary to protect that patient’s privacy. For example, how much and what kind of patient information should be entered into databases. Certainly, the patient has every right to keep their results to themselves. On the other hand, if the patient was identified to possess a VUS that is later reclassified, sufficient information needs to be present so that they may be updated on the status of their variant. In this regard, it has been recommended that certain records within databases only be available to relevant personnel, as is currently executed within the DECIPHER database [14,28]. There are also considerations in circumstances in which the patient’s results may potentially affect another family member. In this situation, the patient has the right to refuse disclosure of their test results to family members who may also possibly be affected.

Most likely, the most significant ethical consideration involves how to handle secondary findings of clinical significance that were not the focus of the original testing. Specifically, these findings would include variants relevant to adult-onset conditions such as breast cancer, Huntington disease, and Alzheimer’s disease, for which the patient is currently asymptomatic [6,28,33]. As a way to manage these types of situations, clinical labs are investigating novel strategies to permit patients to designate what information they would like the clinician to disclose. These strategies would include enhanced pre- and post-test counseling with detailed informed consent on the types of results that can be obtained from this type of testing [6,17]. Informed consent includes implications of positive and or negative/inconclusive results for the patient in addition to legal and insurance implications that would arise with the identification of a pathogenic variant [1,22,35]. Therefore, properly attained informed consent would allow the patient’s family to make an educated decision on what information they want to be divulged. In relation, both physician and lay confusion surrounding results obtained from genomic analysis have been shown to result in insurance discrimination, as well as fear and stigma that serves to compromise the credibility of public health services [35].

Additional problems arise when genetic testing is performed on a child. In these situations, the parents ultimately make the decision on what knowledge to divulge and thus the child is frequently excluded from the decision of whether or not they want to know information that will possibly affect their lives later on. Since the child cannot legally provide informed consent on their own behalf, the parent’s authority should be tempered by the clinician’s obligation to advocate on behalf of the child [1,28]. However, children as young as eight years old can be actively engaged within the testing process to the extent of psychological and cognitive capability that is determined by the clinician in conjunction with the child’s parents [36]. Greater considerations of the child’s desires should be encouraged if the child possesses previous experience in medical decision making as a result of their condition, and/or if they are an adolescent. Both the clinician and the child’s parents should exhibit respect for the child and their associated feelings and opinions whenever applicable. Continued discussions over a period of time have proven to be optimal in cases where the relevance of genomic data can potentially change as novel data and ACMG recommendations arise [36]. Hindering child involvement increases the risk that medical professionals and parents will lose the child’s trust if they later uncover that they were not provided an opportunity to express their feelings in regard to their health. This concurs with previous recommendations that support that upon reaching an age of maturity, minors are able to make informed decisions in regard to their genomic evaluation [35]. 

Future direction of testing

Genomic testing such as CMA and WES have proven to be extremely beneficial in the diagnosis of both inherited and acquired diseases/conditions including DD/ID. It is recommended that clinicians who routinely encounter patients who will require genomic testing, such as patients presenting with DD/ID and associated dysmorphic features, should undergo some form of genetics training, especially in regard to the discovery of VUS. This is hopefully believed to avoid the consequences of mismanagement and/or overtreatment [25]. There is not a sufficient quantity of medical geneticists to hold the primary responsibility of genetic testing and associated counseling [35]. Therefore, primary care providers must have knowledge in regard to the genetic issues that are most probable to affect the populations of patients under their care. This would also include knowledge and expertise on how to adequately prepare families prior to testing. The enhancement of this counseling is a vital issue for pediatric patients recommended for genomic analysis [36]. Additionally, recent reports have established that people of non-European descent are more likely to obtain a result of a VUS [25]. Therefore, additional attention should be focused on the attainment of data from currently underrepresented populations to address this inconsistency of results obtained between varying racial and ethnic groups. In regard to NGS technologies, the main challenge is the establishment of an effective system for data integration for the millions of newly discovered variants in addition to patient information and their associated clinical records [20,33]. The purpose of such a system would ultimately be to efficiently permit the discovery of variants that have been established to contribute to a specific disease/condition. Moreover, it would permit rapid retrieval of pertinent information on millions of variants as well as simplified visualization and assessment guidance [33].

## Conclusions

In conclusion, children presenting with DD/ID and associated dysmorphic features have tremendously benefited from the implementation of CMA analysis. Its significantly higher resolution and lower costs are just a few reasons why it has proven to be superior to other techniques such as G-banded karyotyping and FISH analysis for the diagnosis of conditions such as Angelman syndrome, Prader-Willi syndrome, and VCFS/DGS. However, when CMA is unrevealing, the next best approach is WES analysis for the identification of variants within the human exome. Discovery of a pathogenic variant will allow for enhanced management catered to the patient’s specific needs. On the contrary, the identification of VUS has the potential to create confusion for patients and maybe even some clinicians who lack genetics training during the diagnostic process. There are many considerations, including those of ethical and legal concern, with regard to genetic testing and interpretation of results that still need to be addressed. This is especially accurate in circumstances in which WES is implemented due to the large quantities of data that can be obtained, including that which was not the primary reason of concern that led to genomic analysis. The genetics community has done a suitable job thus far in these regards; however, the previously addressed issues are the remaining challenges that need to be surmounted. In essence, the continued progression of diagnostic genomic testing is expected within the years to come.
